# Asymmetric Omphalopagus in a Triplet after In Vitro Fertilization: A Rare Case of Conjoined Twinning

**DOI:** 10.1155/2018/9349606

**Published:** 2018-08-23

**Authors:** Samir Jabari, Roman Carbon, Manuel Besendörfer, Arndt Hartmann, Oliver Rompel, André Hoerning, Stephan Söder

**Affiliations:** ^1^Institute of Anatomy and Cell Biology, Faculty of Medicine, Friedrich-Alexander-University Erlangen-Nürnberg, Erlangen, Germany; ^2^Department of Pediatric Surgery, Faculty of Medicine, Friedrich-Alexander-University Erlangen-Nürnberg, Erlangen, Germany; ^3^Institute of Pathology, Faculty of Medicine, Friedrich-Alexander-University Erlangen-Nürnberg, Erlangen, Germany; ^4^Department of Radiology, Faculty of Medicine, Friedrich-Alexander-University Erlangen-Nürnberg, Erlangen, Germany; ^5^Department of Pediatrics, Faculty of Medicine, Friedrich-Alexander-University Erlangen-Nürnberg, Erlangen, Germany

## Abstract

**Introduction:**

Asymmetric omphalopagus is a rare situation of conjoined twinning, in which a grossly defective twin is attached to the thorax and upper abdomen of the main twin. We describe a case of an asymmetric omphalopagus accompanied by a normal triplet after assisted reproductive technology (ART) and tried to further characterize the all aspects of the conjoined twins. *Case Presentation*: Perioperative diagnostic imaging was carried out followed by an autopsy to evaluate all aspects of the parasite accompanied by histological, immunohistochemical, and molecular biological evaluation. The parasite had well-developed lower extremities as well as upper extremities with a cleft hand syndrome. The sex was nondeterminable, but DNA fingerprinting revealed that both parasite and autosite are monozygotic, so are females. There was no sign of any axial skeleton or central nervous system. We found a rudimentary rectum with a nonpervious anus, a kidney, ureter, urinary bladder, and a blind-ending urethra. The blood supply of the parasite was connected to the vessel system of the autosite.

**Conclusions:**

To our knowledge, only two cases of parasitic omphalopagus after ART have been described to date. Altogether, 52 cases have been reported, and in most of them, the parasites were successfully separated.

## 1. Introduction

Every report on omphalopagus twins should begin in deference to Chang and Eng, the original Siamese twins, who were born in 1811 and lived to the age of 63. They were symmetrically conjoined omphalopagus twins, connected by a bridge at the level of the liver involving the same with vascular communications in the liver, interconnected xiphoid, but separate peritoneal cavities. Chang died of pneumonia and Eng followed soon after presumably bleeding to death into his brother through the vascular connections they had [1].

Conjoined twinning is a rare condition with a reported incidence of about 1 per 50,000 births [2]. Searching PubMed reveals 157 articles related to the topic (as of May 2018), 156 of them addressing symmetric conjoined twinning. The latest review is from 2017 explaining conception mechanisms, extent of organ sharing, and prognosis of separation [3]. However, just one publication deals with asymmetric conjoined twinning [4]. This is not surprising as asymmetric or heteropagus conjoined twins is an even rarer condition with an incidence of one per one million live births [5]. Heteropagus conjoined twins (also known as parasitic twins) are defined as the presence of a nonviable and severely impaired fetus (or fetal parts) attached to a relatively normal twin (termed the autosite) in one of the same areas in which intact conjoined twins would be united [6]. Either symmetric or asymmetric conjoined twins are thought to derive from either fusion or fission [7]. Eight forms of conjoined twins exist: five types conjoined ventrally and three types conjoined dorsally [8].

For symmetric ventrally conjoined omphalopagus twins, males account for only 29% of the cases, but they account for 77% percent of the cases with parasites. Serious malformations are also seen more often in males making successful surgical separation more unlikely. Frequently associated malformations, for example, are cardiac abnormalities and omphalocele [6].

For surgical separation, it is advisable to remove the parasite completely. However, the surgical approach always poses a challenge because of the sometimes complex features of anatomical fusion [9].

Here, we present a case of an asymmetric omphalopagus female twin joined by a normal triplet after ART, with a fallot tetralogy, an omphalocele, and duodenal atresia, that was successfully separated from the parasite.

This is the first report of an omphalopagus twin after assisted reproductive technology, which was tested with DNA fingerprinting for monozygotic origin, thus supporting the common understanding of the phenomenon.

## 2. Case Presentation

After ART with transfer of three fertilized oocytes, prenatal sonography indicated fetal anomalies and prenatal MRI established the diagnosis of an asymmetric omphalopagus. Because of this diagnosis, the omphalopagus was delivered by cesarean section at 35 weeks of gestation. The omphalopagus was accompanied by a healthy female triplet.

At birth, the omphalopagus had a total weight of 1900 g. The autosite was a completely developed girl with an intact omphalocele containing parts of the liver. The umbilical cord contained only two vessels.

**Figure 1:**
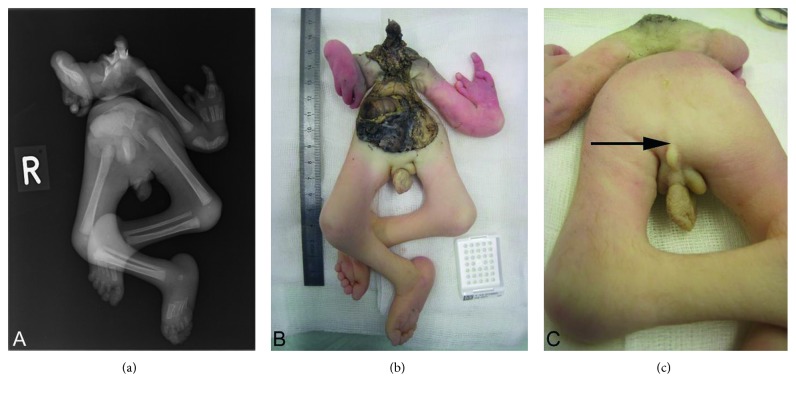
(a) Conventional radiology and (b, c) macroscopic presentation upon autopsy. Note the nonpervious anus depicted with an arrow on (c).

On her left thorax, a parasitic twin was attached (Figure 1). It displayed well-developed lower and upper extremities with a rather hypoplastic right arm and less hypoplastic left arm with a cleft hand syndrome and a tetra dactyly, respectively. No spontaneous movement of the parasite's limbs was observed after birth. At the pelvis, a skin cleft suggested a scrotum, but no testes were found. Furthermore, anal atresia was found and spontaneous micturition occurred.

**Figure 2:**
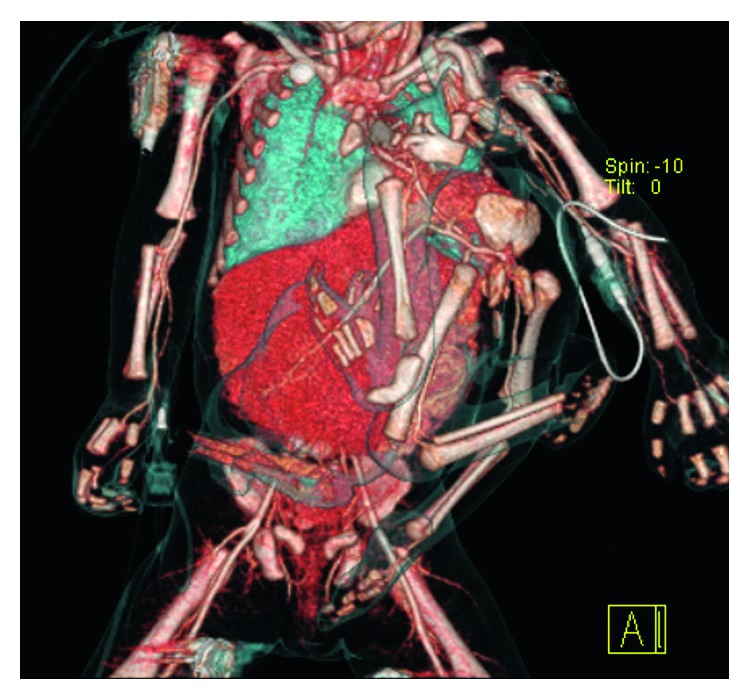
Three-dimensional CT reconstruction of presurgical presentation. Note the ventral vessel deriving from the aortic arch pulling into the parasite and the retrograde proceeding vessel into the autosite via the omphalocele as in the falciform ligament.

3D CT and MRI (Figure 2) showed that the autosite had a fallot tetralogy and a double aortic arch with its ventral branch splitting into a large vessel: one leading to the parasite (forming the equivalent of an aorta there) and the second vessel inserted into the omphalocele forming the superior mesenteric artery. In the autosite, an atresia of apple peel type and a malrotation of the intestine were detected. The omphalocele contained one halve of the circumference of the autosite's liver.

**Figure 3:**
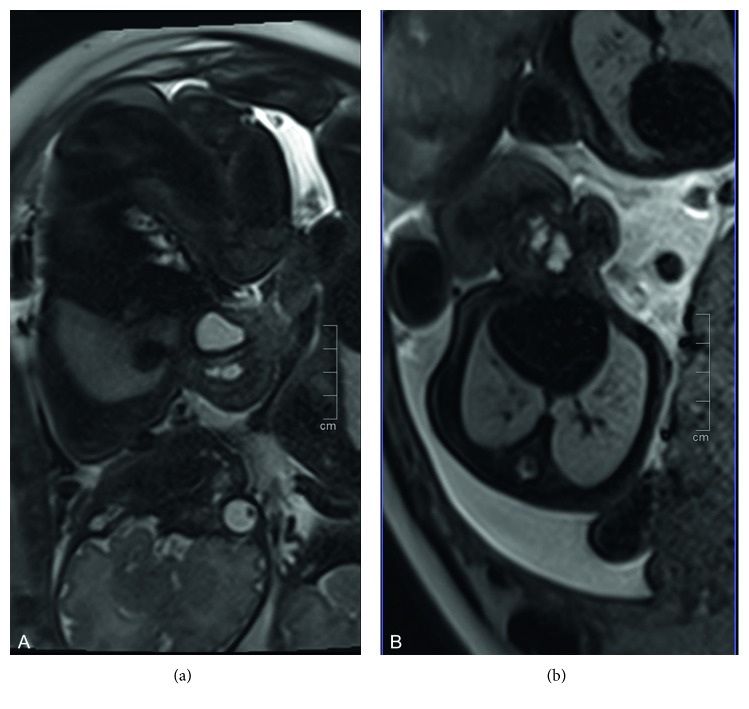
Preterm MRI depicting a ventral corpus to the autosite on the right in sagittal view (a). Same situation in cross-sectional plane (b).

The parasite displayed a fusion of the shoulder girdle consisting of a dysplastic scapula and clavicula connected to the sternum and left chest and a fusion of the rather well-developed pelvis to the lower sternum and left chest. In the parasite, no spine was detected. But a single kidney and a urinary bladder filled with urine indicate functional urine transport (Figure 3). No ovaries or testes were found in the parasite's pelvis. The large vessel from the autosite branched into the iliac arteries. No head, lung, or other organ systems were detected in the parasite.

The separation was planned and conducted by an interdisciplinary team including general pediatric surgery, heart and thorax surgery, urology, neonatology, pediatric cardiology, gastroenterology, neurology, and radiology, three days after the cesarean section.

Initially, the shoulder girdle of the parasite was separated from the thorax of the autosite. The large arteria originating from the ventral branch of the doubled aorta inserting into the parasite was excavated and sealed with a clip. Next, the chondroid fusion between left upper region of the sternum and ribs III to V and the dysplastic scapula and clavicula was separated. The continuity of the sternum and ribcage was preserved.

The second step was the resection of the omphalocele at the border between Wharton's jelly and skin. A narrow connection between the peritoneal cavity of the autosite and a rudimentary peritoneal cavity of the parasite was found. The atretic duodenum together with attached parts of the joint mesenterium as well the appendix vermiformis was dissected. The peritoneal cavity and a minor defect in the left part of the diaphragm/pericardium were sealed using the available peritoneum of autosite and parasite as well as a 4 by 5 cm patch (Tutopatch, Bess Medizintechnik Gmbh, Berlin, Germany). Minor ruptures of the capsule of liver and spleen occurred in the process of relocation of the organs and were treated successfully by sutures and/or fibrin sealant patches (TachoSil, Takeda, Berlin, Germany).

Then, the pelvic portion of the parasite was separated from the autosite's thorax inserting the ribs VII to IX. Where the continuity of the rips could not be fully preserved, the defects were closed by reconstructive surgery. After complete removal of the parasite, the autosite's abdominal wall was reconstructed.

At the age of three and seven months, cardiovascular surgical treatment of the fallot tetralogy was successfully performed in a two-step approach.

**Figure 4:**
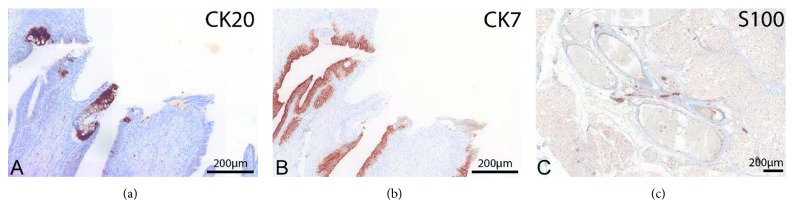
(a, b) Immunohistochemical staining of intestinal tissue showing immunoreactivity for CK20 (a) and CK7 (b) showing intestinal expression pattern. (c) The picture on the right depicts nervous tissue in the body immunoreactive for S100.

The parasite measured 19 cm at the greatest distance with the rump length of 11 cm and had a weight of 167 g. It had well-developed lower extremities and pelvis as well as upper extremities with a cleft hand syndrome and syndactyly seen in the radiography (Figure 1). From the outer aspect, the sex was nondeterminable, except it had a scrotum-like skinfold, but we could not find any testis or ovaries. A SRT analysis (DNA fingerprinting) with tissue of autosite and parasite showed no differences, indicating that both parasite and autosite are monozygotic and therefore both share female genotype. There was no sign of any axial skeleton or central nervous system. However, we could clearly detect nerve plexus at the upper extremities in the location of the plexus brachialis as well as peripheral nerves within the whole body (positive for S100 in immunohistochemical staining, Figure 4). In the pelvis, we found a rudimentary rectum with a nonpervious anus (with mucosa immunohistochemically positive for CK7 and CK20, Figure 4), a kidney, ureter, urinary bladder, and a blind-ending urethra. We found cartilage, normally developed bone, and bone marrow.

The vascular connection to the autosite was through a double aortic arch and a prominent artery starting from it, similar to an A. thoracica int., which centrally tangles the condensed shoulder girdle and pulls at the posterior trunk wall (resembling the aorta) into the pelvis of the parasite, where it forms the iliac bifurcation (Figure 2). Furthermore, the prominent artery gives off a strong branch to the omphalocele, which later turns into an A. mesenteric sup. in the mesenterium commune of an apple peel small intestine with atresia and muzzle in the falciform ligament.

## 3. Discussion

As this is the first report to clearly show monozygotic origin of omphalopagus twinning after in vitro fertilization, we want to point out and review the importance and general aspects of this phenomenon in the following.

All conjoined twinning, symmetric or asymmetric, is thought to derive from “fusion” of two separate monozygotic embryonic discs but unlikely derived from an incomplete “fission” of the blastocyst [7]. According to the “spherical” theory, the two originally separate embryonic discs fuse always homologously either dorsally at the closing neural folds or ventrally at structures surrounding a common yolk sac. Eight different types of conjoined twins can be distinguished: five ventrally united and three dorsally united types. The fusion occurs at a very early embryonic stage at around the third to fourth week, resembling the embryonic disc stages. The fusion takes place only at sites designated to fuse in order to create an enclosed body and never at places of an already closed body or cavity. The dorsally conjoined twins fuse with the closing neural folds either in the head (craniopagus), the coccygeal region (pyopagus), or in between these regions (rachipagus). The ventrally united twins may fuse at the oral end of the disc at the level of the septum transversum, the cardiogenic region, or the oropharyngeal membranes resulting in omphalopagus, thoracopagus, or cephalopagus. They can also fuse at the caudal end of the disc at the site of the cloacal membrane resulting in the so-called ischiopagus twins. The last possible fusion site for ventrally conjoined twins is the margin of the embryonic disc resulting in a parapagus [9].

Asymmetric twins may result from the demise of one twin with the surviving of supernumerary structures attached to the more normal twin. The sites of the union are the same as described above. The more defective one is called parasite and the other autosite. Most often the parasite contains no or only a rudimentary heart or neural tube structures [6].

Omphalopagus asymmetric twinning is an extremely rare condition, with only 52 cases published to date with parasites resembling an almost complete but headless infant or a parasite consisting only of a supernumerary limb [4, 9]. Most of the omphalopagus parasites however have upper and lower limbs, the lower most often almost normal. Rudimentary genitalia are common and usually associated with kidneys. Anal dimples are seen but are almost always nonpervious [9].

As the omphalopagus is thought to share a common yolk sac, it is not surprising that they sometimes share parts of the peritoneal cavities and the intestinal structures.

The absence of the brain and heart in almost all cases (except for eight) may not be incidental. A defective heart may be the cause for the demise of the defective twin and absence of the brain. The existence of nerves however shows that an initial development of neuronal structures takes place [6].

The autosite sometimes has malformations not associated with the union as malformations of the heart such as fallot tetralogy, but these malformations are the same as found in singletons [9].

In two cases, a normal triplet accompanied the twins; four cases of omphalopagus twins after assisted reproductive technology have been described to date; however, only one of them was an asymmetric omphalopagus twin [10].

All conjoined twins are thought to be monozygotic, but there are new reports indicating doubts about this widely accepted premise [11]. In our case, there is no indication of a fusion of dizygotic twins.

Perinatal ultrasonographic examination or magnetic resonance imaging (MRI) should early be used for diagnosis and planning of the multidisciplinary treatment procedure [12, 13].

Because the associated malformations of heteropagus twins are very complex and have a very broad spectrum, an interruption of pregnancy solely based on the diagnosis of heteropagus twinning seems not to be completely justifiable and should be decided individually.

In fact, the presence and degree of the severity of cardiac malformations as pointed out above may be the determinant factor for the prognosis.

The present case shows that preoperative radiologic picturing used to portray the internal structures of the parasite and its interconnection with the autosite is a helpful tool in indicating the position of major vessels and hereby a vital part for the surgical approach.
